# A Laboratory Investigation of the Effectiveness of Different Acids in Removing Mineral Trioxide Aggregate From Simulated Root Canals

**DOI:** 10.1111/aej.12937

**Published:** 2025-03-24

**Authors:** Stephanie Chan, Paul V. Abbott

**Affiliations:** ^1^ UWA Dental School The University of Western Australia Perth Australia

**Keywords:** acids, endodontic re‐treatment, HCSC, MTA, rotary files, ultrasonics

## Abstract

The aim was to compare the effectiveness of acids in removing MTA from root canals. Fifty acrylic blocks were filled with MTA and divided into groups – 10% hydrochloric acid; 10% citric acid; 2% acetic acid; 2% carbonic acid and 20% tartaric acid. MTA was removed with the acids, plus ultrasonic tips and rotary files. Outcomes were measured as pre‐ and post‐operative weight differences, luminance differences and working time to regain length or form ledges. Weight differences varied significantly – citric acid was highest; hydrochloric acid was lowest and others were not statistically different from each other. Luminance did not differ significantly between groups but varied significantly with distance from the apex – hydrochloric acid and carbonic acid were lowest at 1 mm from the apex. Working times differed significantly – hydrochloric acid took longer than other acids except citric acid. Overall, the most effective acid was citric acid. The least effective was hydrochloric acid.

## 1 Introduction

One of the cornerstones of successful root canal treatment is the quality of the root canal filling [1, 2, 3]. A well‐filled root canal is thought to prevent bacteria and their by‐products from reaching the periapical tissues and conversely serves to also prevent any nutrients in the periapical tissue from reaching and nourishing any intracanal bacteria, hence preventing re‐infection of the root canal system [4]. Historically, the most widely used core material for the root canal filling has been gutta percha (GP) which has been used with a variety of root canal cements [5, 6]. However, studies have shown that GP/cement root canal fillings alone do not prevent the penetration of bacteria through tooth roots regardless of the root filling technique used [4, 6, 7].

In recent decades, hydraulic calcium‐silicate cements (HCSCs), notably mineral trioxide aggregate (MTA), have emerged with promising physicochemical and biological properties in comparison to GP. These include superior sealing capabilities (even in the presence of blood and moisture), minimal microleakage, biocompatibility, the ability to induce and be conducive to hard tissue formation and promotion of periodontal ligament regeneration [8]. Additionally, when compacted against dentine, HCSCs form an adherent interstitial dentine‐HCSC layer which resembles hydroxyapatite in composition and structure, and this gives HCSCs their high marginal adaptation and sealing properties [9, 10]. Given these characteristics, HCSCs have been promoted and used in a variety of endodontic settings such as root canal fillings [8, 11].

However, despite HCSCs superior sealing abilities, studies have shown that teeth filled with HCSCs are still susceptible to bacterial penetration and thus these materials cannot provide a complete barrier against bacteria [12, 13]. Hence, re‐infection of the root canal system due to subsequent bacterial re‐colonisation remains possible even with HCSCs, and clinicians may need to remove and re‐treat the root canal system. Unlike GP, which can be removed relatively easily and effectively with solvents and instruments, thus far, no method has been reported for the removal of HCSCs from the root canal once it has set. This is likely to be the biggest problem associated with HCSCs, as it may deem the tooth unsalvageable if re‐infection were to occur, or endodontic surgery may be required.

Current studies have investigated a range of chemical solutions and mechanical instrumentation techniques to address these challenges and whilst no single approach achieves complete removal, combinations such as rotary files and ultrasonic tips show potential [14]. Solutions such as carbonic acid, acetic acid and hydrochloric acid have shown promise in reducing MTA surface hardness [15, 16, 17, 18, 19, 20] yet their efficacy in complete dissolution varies as existing studies have presumed that decreasing the microhardness of MTA would allow it to be more easily removed since microhardness is an indication of a material's resistance to deformation [18, 19]. Whilst there may be some correlation between a substance's microhardness and its ease of removal, this presumption has not been substantiated within the literature. To date, no single experimental method has been reported to be effective in assessing the ability of a technique, solvent or instrument to remove MTA [18]. Additionally, there is no study that has examined the simultaneous application of instruments (e.g., files and ultrasonic tips) and acids in removing HCSCs. This would be of high interest as it has been reported that instruments and various acids when used alone only had partial success in the removal of MTA.

The aim of this study was to evaluate the use of different acids concurrently with ultrasonic tips and rotary re‐treatment files to establish effective protocols for the removal of set HCSCs from root canals. The hypothesis was that one or more of the tested acids would aid the removal of MTA from simulated canals when used with ultrasonic tips and rotary re‐treatment files.

## 2 Materials and Methods

### 2.1 Sample Preparation

The sample size for each group was determined using an online statistical tool (https:/clincalc.com/stats/samplesize.aspx). The study was a two independent sample study with a dichotomous endpoint (working length reached or not reached). With an *α* value of 0.05 and power of 80%, the sample size required was 10 for each experimental group.

Fifty transparent acrylic blocks with 10° curved canals (Nissin, Kyoto, Japan) were accessed initially with an ISO size 15 Hedström file (Kerr Dental, Orange, CA, USA) to determine a working length of 20 mm. Each canal was then instrumented with ProTaper Next (Dentsply Maillefer, Ballaigues, Switzerland) NiTi rotary files sizes X1 (17/04), X2 (25/06) and X3 (30/07) whilst using 15% EDTA‐C (15% EDTA and 0.85% Cetrimide; DentaLife Pty. Ltd., Ringwood, VIC, Australia) as an irrigant between each file.

The canals were then dried with paper points (Dentsply Maillefer, Ballaigues, Switzerland), and the blocks were left at room temperature for 24 h to ensure complete drying. Each instrumented block was then weighed to the nearest 0.001 g using a digital electronic balance (Sartorius Entris 323‐1S Analytical Digital Electronic Balance, Sartorius, Aubagne, France).

The root canals were filled with ProRoot MTA White (Dentsply Tulsa Dental, Oklahoma, USA) using the Lawaty technique. This technique has been described by Bogen & Kuttler [9] and Mathew et al. [21]. The technique involves using the number 1 NiTi memory shape needle (and its corresponding plastic piston) of the Micro Apical Placement system (Produits Dentaires SA, Vevey, Switzerland) to deliver the ProRoot MTA into the canals. Then, K‐files (Maxima, Henry Schein, Germany) are used to pack the MTA starting with an ISO size 25 file in the apical third of the canal, and then, progressively larger files are used towards the coronal aspect via a ‘step‐back’ method after which a final vertical compaction with a size one (red) Buchanan Hand Plugger (Kerr Dental, California, USA) is performed. In this study, the coronal 3 mm of each canal was left unfilled to provide a reservoir for the later insertion of the acids during the re‐treatment procedure.

**FIGURE 1:**
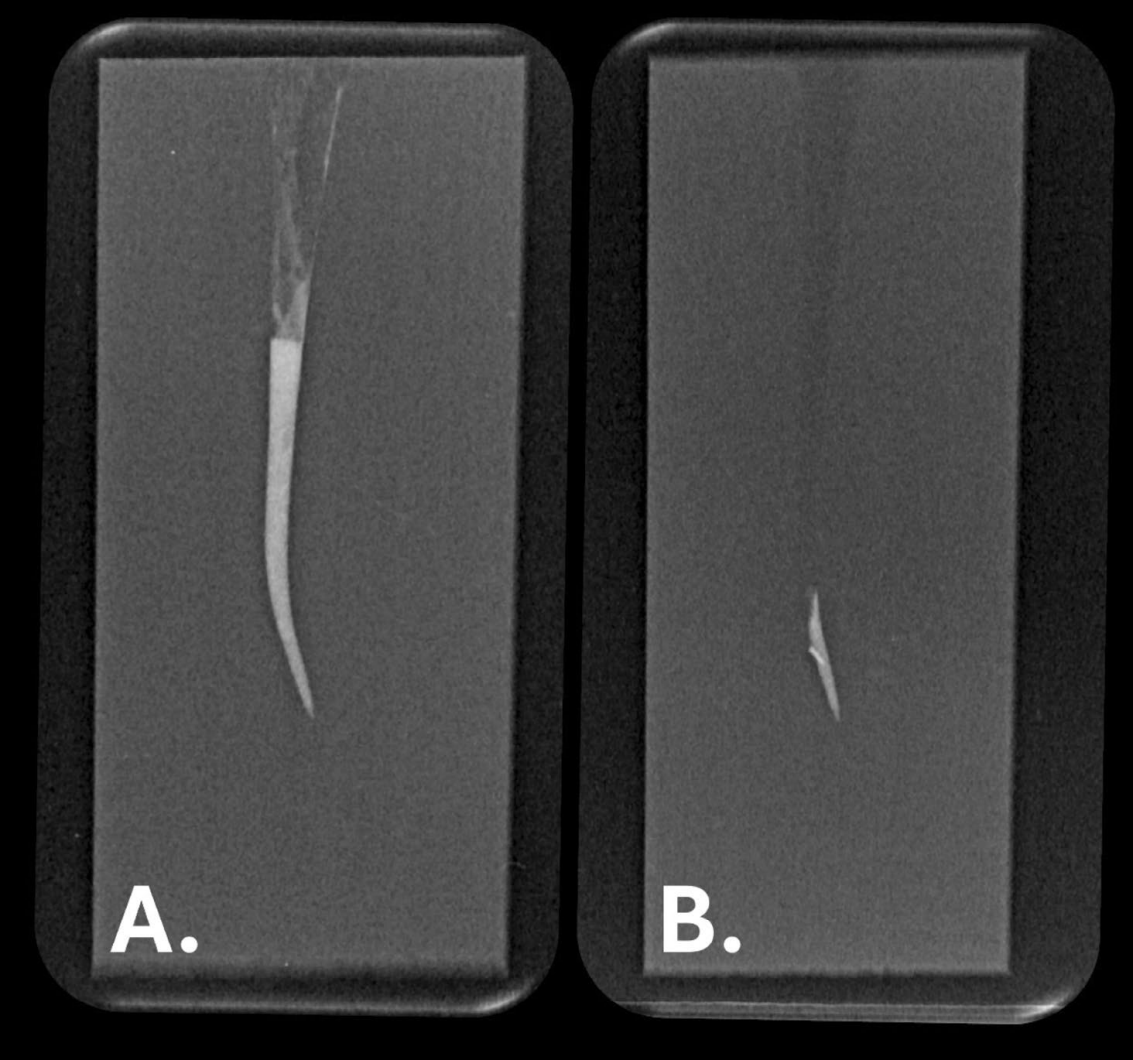
Representative radiographs of plastic blocks: (A) Following filling of the canal with MTA and (B) after attempted removal of the MTA root canal filling.

Samples were then incubated in 100% relative humidity at 37°C for 10 days to allow complete setting of the MTA. Afterwards, all samples were numbered, weighed again and a periapical radiograph was taken to check for the quality of the root canal filling (Figure 1A).

### 2.2 Re‐Treatment

Samples were then randomly allocated to five experimental groups (*n* = 10) according to the chemical acid used:

- Group 1%–10% hydrochloric acid (HCl)
- Group 2%–10% citric acid (CA)
- Group 3%–2% acetic acid (AA)
- Group 4%–2% carbonic acid (CAA)
- Group 5%–20% tartaric acid (TA)

The preparation of all acids at the required concentrations was carried out in the School of Molecular Sciences, University of Western Australia, Western Australia.

For each group, two drops of the corresponding acid were placed in the unfilled coronal reservoir of each sample for three minutes. An attempt to re‐establish the working length was then done initially using an Acteon Newtron scaler handpiece (Satelec Acteon, Mérignac, France) with an ET20D ultrasonic tip (Satelec Acteon, Mérignac, France) in the coronal third of the canal, followed by an ET25 ultrasonic tip (Satelec Acteon, Mérignac, France) in the middle third of the canal at 55% of maximum power. A ProTaper Universal Retreatment (Dentsply Maillefer, Ballaigues, Switzerland) rotary file size D3 (20/07v) was then used at a constant speed (600 rpm) and torque (2.5 N/cm) in the apical third of the canal. During this re‐treatment process, there was continual replenishment of the acid, which was delivered via a syringe. The time taken for the re‐treatment process was also recorded–this was designated as the ‘working time’.

**FIGURE 2:**
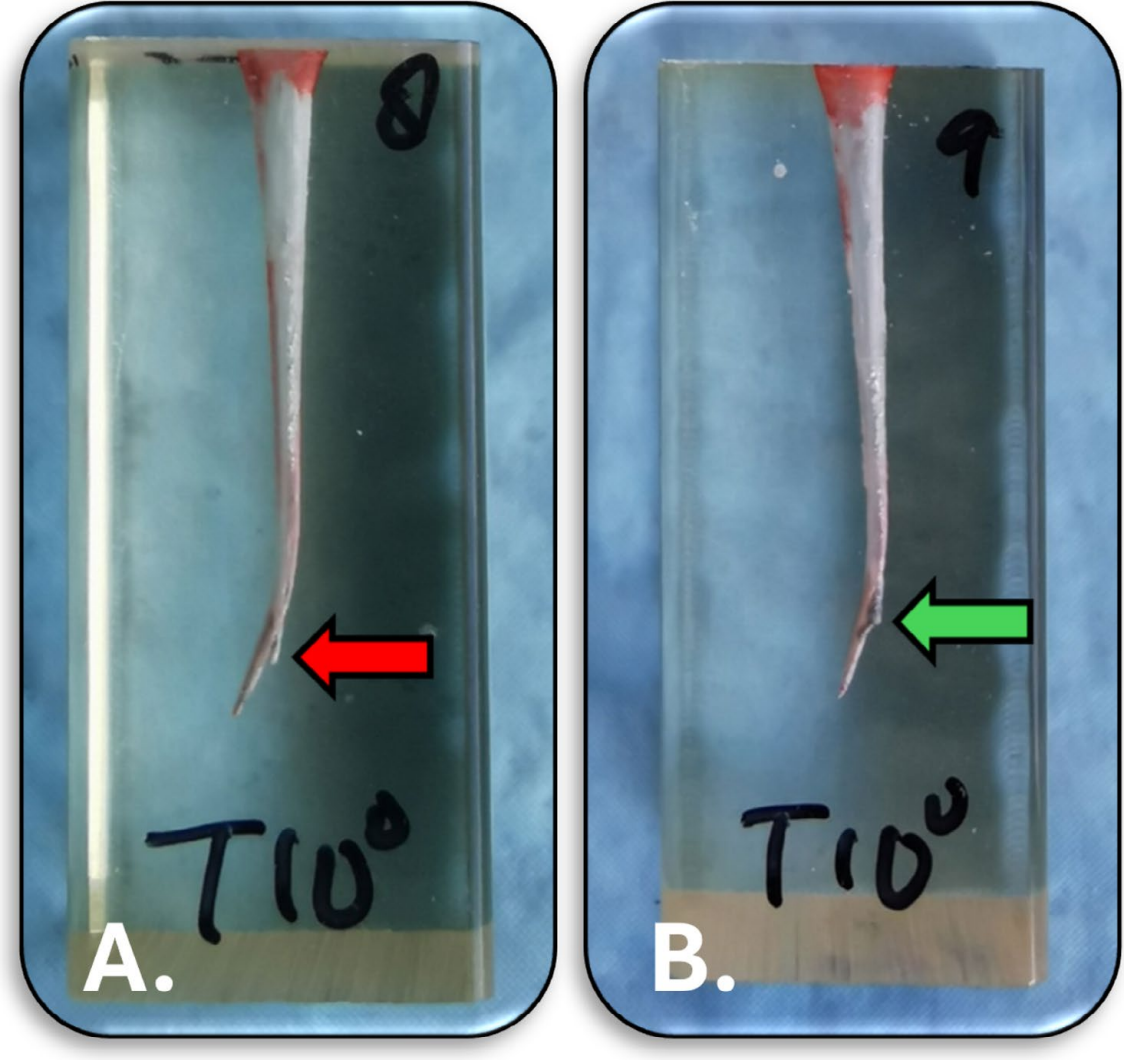
Sample showing: (A) Ledging (red arrow) of a canal at the start of canal curvature and (B) a fractured ProTaper Universal Retreatment D3 rotary file (green arrow) at the start of canal curvature. Images taken with a Huawei P30 Pro camera.

The re‐treatment process was deemed complete when the file reached the full working length, an iatrogenic error such as a ledge or a file fracture occurred (Figure 2), or when the working length could not be achieved after a period of 30 min. In cases where the working length was reached, this was checked and confirmed with a size 15 Hedström file. A final rinse of the canal was done with 5 mL of 0.9% saline solution (Baxter, NSW, Australia).

A new rotary file was used after every five samples or when a file was noted to be distorted or showed signs of fatigue. A new ultrasonic tip was only used if the existing tip fractured.

The canals were dried with paper points, and the blocks were stored at room temperature for 24 h. They were then weighed again, and a periapical radiograph was taken to assess whether any MTA remained in the canal (Figure 1B).

All stages of the experiment were conducted by a single operator who was an experienced clinician with advanced endodontic training.

### 2.3 Evaluation of MTA Removal

The removal of MTA was evaluated with three methods:

1. The MTA mass difference of each block's filled and retreated weight.
2. The radiographs taken after root canal filling and after removal of the MTA were exported in JPEG format (Figure 1A,B, respectively). The radiopacity of the images for each sample was compared by measuring luminance within the canals at 1 mm, 5 mm and 10 mm from the apical end of the canal. Luminance was calculated with the average image whiteness measured using an online calculation tool (https://planetcalc.com/7779/).
3. The ability to reach working length was determined for each sample, along with the time taken.

Numerical data were presented as mean, standard deviation (SD), median, minimum and maximum values. They were explored for normality and variance homogeneity by checking the data distribution and by using Shapiro–Wilk's and Levene's tests, respectively. Weight difference data were normally distributed, but the homogeneity assumption was violated, so they were analysed using Welch one‐way ANOVA followed by the Games‐Howell post hoc test. Other data were non‐parametric and were analysed using the Kruskal–Wallis test followed by Dunn's post hoc test for intergroup comparisons and Friedman's test followed by Nemenyi's post hoc test for intragroup comparisons. The P‐values were adjusted for multiple comparisons utilising The False Discovery Rate (FDR) method. The significance level was set at *p* < 0.05. The statistical analysis was performed with R statistical analysis material version 4.3.3 for Windows.

## 3 Results

The full working length was reached in nine of the 50 canals. Five canals were blocked with MTA that could not be removed within 30 min, 34 canals had ledges, and two canals had both a ledge and a fractured file.

### 3.1 MTA Weight Difference

The mean differences in mass (and 95% CI) between the filled samples and after MTA removal are shown in Table 1. Intergroup comparison indicated a significant difference between values in the different groups (*p* = 0.017). Post hoc pairwise comparisons showed that the 10% CA group had a significantly higher value than the 10% HCl group (*p* < 0.05). No significant differences were found amongst the remaining groups (Table 1).

**TABLE 1:** Summary statistics and intergroup comparison for weight difference before and after removal of MTA.

Weight difference (mg) (Mean ± SD)					Test statistic	*p*
10% Hydrochloric acid	10% Citric acid	2% Acetic acid	2% Carbonic acid	20% Tartaric acid		
15.80 ± 2.30^B^	21.20 ± 5.45^A^	19.50 ± 2.51^AB^	17.20 ± 2.30^AB^	17.80 ± 2.35^AB^	3.80	0.017*

*Note:* Values with different superscripts within the same horizontal row are significantly different.

Abbreviation: SD, standard deviation.

* Significant (*p* < 0.05).

### 3.2 Relative Luminance

**FIGURE 3:**
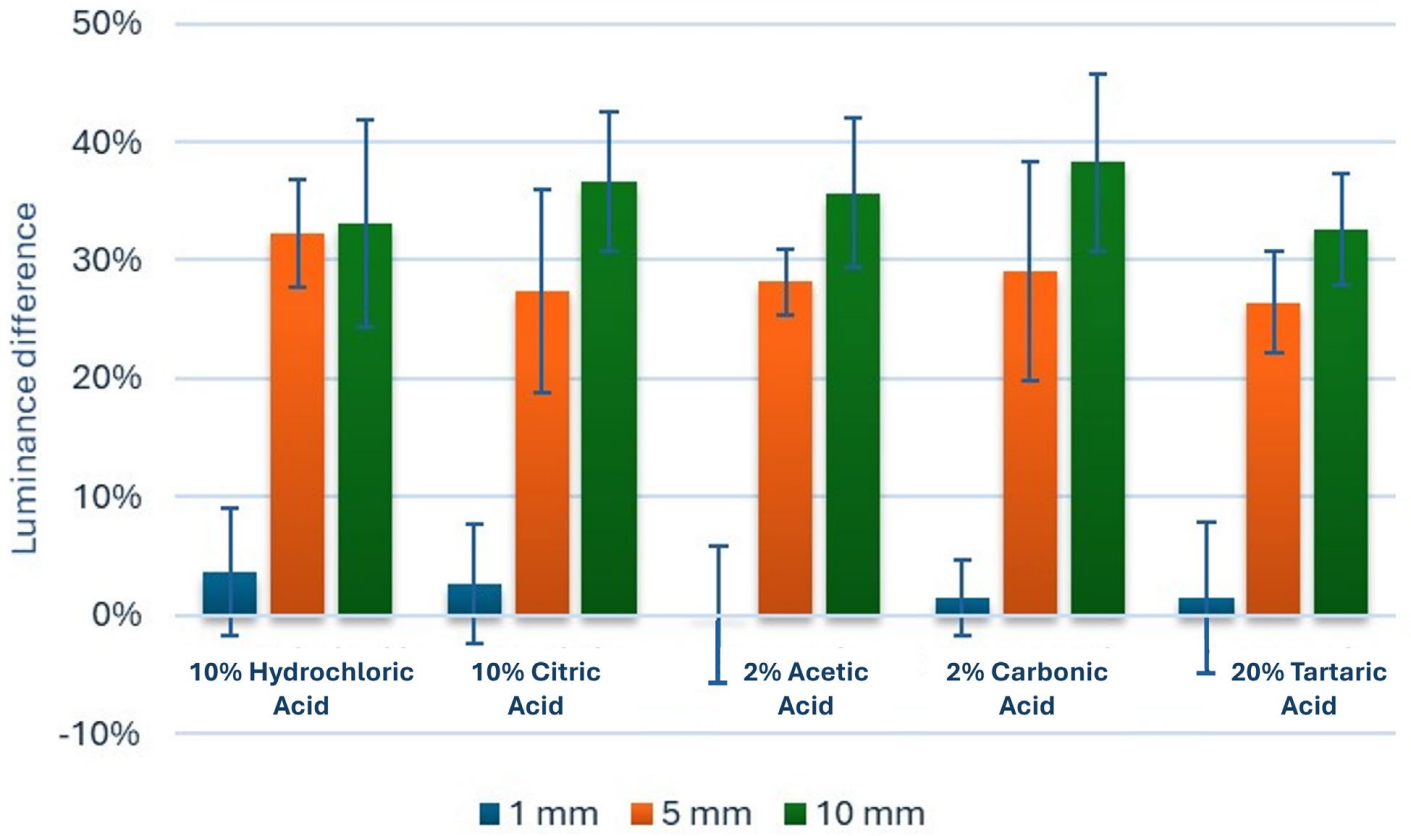
Bar chart showing mean and standard deviation (error bars) values for the difference in luminance (%).

Estimated mean values for each group's relative luminance (radiopacity) at each depth (1 mm, 5 mm and 10 mm from the apical end of the canal) are presented in Figure 3. The intergroup comparisons (Table 2) indicate that regardless of the distance from the apical end of the canal, there was no significant difference between tested groups (*p* > 0.05). However, within all groups, there was a significant difference between the values measured at different distances (*p* < 0.005). For the 10% HCl and 2% CAA groups, post hoc pairwise comparisons showed values measured 1 mm from the apical end of the canal were significantly lower than those measured at the other distances (*p* < 0.001). For all other groups, they were all statistically significant (*p* < 0.001), with the highest values measured at 10 mm from the apical end of the canal, followed by 5 mm, and the lowest values were measured at 1 mm from the apical end of the canal.

**TABLE 2:** Summary statistics, intergroup and intragroup comparisons for the difference in luminance.

Distance from the apical end of the canal	Luminance difference (%) (Mean ± SD)					Test statistic	*p*
	10% Hydrochloric acid	10% Citric acid	2% acetic acid	2% Carbonic acid	20% Tartaric acid		
1 mm	3.65 ± 5.44^Ab^	2.67 ± 5.03^Ac^	0.11 ± 5.72^Ac^	1.46 ± 3.22^Ab^	1.50 ± 6.30^Ac^	3.28	0.513
5 mm	32.28 ± 4.51^Aa^	27.40 ± 8.60^Ab^	28.16 ± 2.72^Ab^	29.08 ± 9.30^Aa^	26.45 ± 4.27^Ab^	8.48	0.228
10 mm	33.12 ± 8.81^Aa^	36.63 ± 5.93^Aa^	35.64 ± 6.32^Aa^	38.24 ± 7.42^Aa^	32.61 ± 4.72^Aa^	4.85	0.454
Test statistic	15.20	16.80	18.20	15.80	18.20		
*p*	< 0.001*	< 0.001*	< 0.001*	< 0.001*	< 0.001*		

*Note:* Values with different upper and lowercase superscripts within the same horizontal row and vertical column, respectively, are significantly different.

Abbreviation: SD, standard deviation.

* Significant (*p* < 0.05).

### 3.3 Time Taken for MTA Removal

The mean working times taken for the removal of MTA (95% CI) are listed in Table 3. This Table also shows intergroup comparison for the working times, which indicates a significant difference between values measured for the different groups (*p* < 0.001). Post hoc pairwise comparisons showed the time measured for 10% HCl was significantly longer than all other groups except for 10% CA (*p* < 0.05). Additionally, the time measured for 10% CA was significantly longer than 20% TA and 2% AA (*p* < 0.05), plus the time measured for 2% CAA was significantly longer than 2% AA (*p* < 0.05).

**TABLE 3:** Summary statistics and intergroup comparison for the time taken for MTA removal.

Time taken for MTA removal (min) (Mean ± SD)					Test statistic	*p*
10% Hydrochloric acid	10% Citric acid	2% Acetic acid	2% Carbonic acid	20% Tartaric acid		
11.06 ± 3.16^A^	9.12 ± 1.22^AB^	6.26 ± 1.14^D^	8.18 ± 1.35^BC^	7.28 ± 0.77^CD^	31.21	< 0.001*

*Note:* Values with different superscripts are significantly different.

Abbreviation: SD = Standard deviation.

* Significant (*p* < 0.05).

## 4 Discussion

The present study investigated the efficacy of various acids and instruments on the removal of MTA from simulated root canals. Analysis of the mean difference in mass across the five acid groups revealed distinct patterns of efficacy. Notably, the 10% HCl group had the lowest mean mass difference of 15.80 mg, indicating the least amount of MTA removed from the root canal space compared to the other acids. In contrast, the 10% CA group had the highest value of 21.20 mg, suggesting greater efficacy in MTA dissolution and removal.

This finding contrasts the results of previous studies [17, 20] where, despite 10% CA and 20% TA significantly affecting the microhardness of MTA, 2% CAA was found to have the greatest impact. A possible explanation for these variations could lie in the different methodologies employed to assess the solvent efficacy on MTA. In the studies by Butt & Talwar [17] and Abraham et al. [20], solvent efficacy was determined by examining its effect on the microhardness of MTA. However, there is no reported correlation between microhardness and a material's ease of removal. Whilst microhardness measurements provide valuable insights into the mechanical properties of materials such as MTA, they do not necessarily reflect their behaviour during removal procedures. Factors such as chemical dissolution, adhesion and mechanical disruption during removal may significantly influence clinical outcomes. In contrast, the current study evaluated acid efficacy by removing the MTA root canal filling in conjunction with the use of files and ultrasonic tips. This approach provided a more direct assessment of an acid's ability to dissolve and remove MTA from the root canal space, removing potential confounding factors associated with microhardness measurements.

Of the five acids tested in this study, 10% HCl was the least effective in removing MTA from the simulated root canals. This was surprising, especially considering the widespread use of HCl in the construction industry for the dissolution of cements such as Portland cement. However, the choice of concentration in this study may be a factor that influenced the effectiveness of HCl. Two previous studies [18, 22] used higher concentrations of HCl (37% and 20%, respectively). The lower concentration used in the current study was influenced by observations from Rezaei et al. [22] who reported that 20% HCl resulted in significant erosion of the dentine tubule orifices. Whilst higher concentrations of HCl may offer enhanced dissolution capabilities, they also pose a greater risk of adverse effects on the surrounding dentine.

The effect of concentration is further highlighted by the finding that the 10% HCl group had the longest mean time for the re‐treatment procedure (11.06 min). This extended working time suggests potential challenges in effectively dissolving MTA with this concentration. In addition, the sequence in which samples were tested, with the HCl and CA groups being retreated first, could have influenced the observed mean working times, as it was noted that 10% CA also exhibited a significantly longer working time compared to the other acids. Clinician experience may have influenced the outcome, given the mean working time was noted to decrease with subsequent groups, underscoring the importance of considering both concentration and testing protocol in this study.

It is important to consider the influence of the MTA setting time on the study outcomes. Based on the study by Boutsioukis et al. [14], MTA was left to set for 10 days before re‐treatment was attempted. Whilst no explanation was offered by Boutsioukis et al. [14] for selecting this specific time, one possible explanation could be that 10 days was chosen to allow for some development of MTA's physical properties following its initial setting time of four hours. This additional time might have been deemed necessary to enable partial maturation of MTA, including enhancement of its compressive strength and adhesion to the root canal walls. However, it is important to note that MTA may require up to 21 days to achieve complete setting and to attain its maximum compressive strength [9]. Using a setting time of 10 days in the current study potentially resulted in incomplete maturation of the MTA which may have had implications for its removal characteristics. The shorter maturation time may have rendered the MTA more susceptible to dissolution and mechanical disruption since MTA that has not fully matured may have reduced adhesion to the root canal walls and diminished compressive strength, making it easier to remove. Consequently, this could create a false impression of an acid's efficacy in removing MTA, as the reduced time for setting may have facilitated its removal. Despite this, the same setting time was used for all groups so any such effect would apply to all of the acids that were tested.

The current study included a novel approach by utilising pre‐operative and post‐operative radiographs (Figure 1) to assess the removal of MTA from the root canals through an examination of luminance values. This was based on the study by Mathew et al. [21] who used radiographs to determine how densely packed the root canal fillings were when filled with MTA using different compaction techniques. They used relative luminance to evaluate the radiographic density of digital images by calculating the average image whiteness (i.e., radiopacity) along the filled root canal. This was achieved by measuring each assigned increment at a standardised location along the canal. In the current study, any contrast difference between images was taken to indicate the removal of the MTA.

The current findings revealed successful removal of MTA from the coronal and middle root thirds across all acid groups, as indicated by substantial luminance difference values recorded at distances of 10 mm and 5 mm from the apical end of the canal. However, in the apical third of the canals, MTA removal was more challenging for all acid groups. This difficulty was underscored by the minimal luminance differences observed at 1 mm from the apex. The inability to remove MTA from the apical region likely stems from the 10° curvature present in the root canal models, which prevented the use of ultrasonic tips and rigid rotary re‐treatment files beyond the start of the curve. Models with this curvature were chosen to simulate the very common clinical situation of apical curvatures in tooth roots. Attempts to reach the full working length usually resulted in iatrogenic mishaps such as ledging of the canal or fracture of a rotary file (Figure 2). Patency could only be achieved in a small number of samples, which may be attributed to inadequate compaction of the MTA root filling, the existence of voids at the curvature's junction (which may have facilitated easier penetration of the rotary re‐treatment file), or because the acids were ineffective in dissolving or softening the MTA. It may also have been related to the size of the re‐treatment file (20/07) being smaller than the size of the final file used when originally preparing the canals (30/07).

These findings and challenges are similar to those of Boutsioukis et al. [14] who reported that achieving patency was contingent upon the ultrasonic tips reaching the working length, but this could not be consistently achieved, despite them exclusively using teeth with straight root canals. Even in cases where patency was attained, remnants of MTA persisted on the canal walls, leading to the inference that complete removal of MTA from the root canal was unattainable. Ng et al. [23, 24] have reported that being able to reach the apical foramen and obtaining apical patency is a significant predictor of endodontic treatment outcomes. The results of the current study show that reaching the apical end of the canal when re‐treating canals that have been completely filled with MTA is problematic and therefore the outcome of non‐surgically re‐treating such canals is likely to be affected and less predictable.

Whilst two‐dimensional (2D) imaging was used in this study to assess radiographic density in order to simulate the clinical scenario, it may have also underestimated the amount of MTA remaining on the root canal walls. Some studies have used micro‐CT imaging for volumetric analysis, specifically to measure voids within MTA root canal fillings in extracted human teeth [25, 26, 27] but to the authors' best knowledge, its use in acrylic root canal models has not been reported. Micro‐CT imaging may offer a more accurate assessment of the root canal filling and its removal, and it could possibly be employed in future studies.

The technique used to fill the canals was the Lawaty technique as described by Bogen & Kuttler [9] and Mathew et al. [21]. The latter study assessed four different techniques to determine which method was the best to fill canals with MTA. They concluded that whilst the Lawaty technique took the longest time, it was also the method that produced the highest mass and greatest radiopacity of the root canal filling. In addition, since this technique is being used clinically [9], there is some relevance to the clinical situation and this technique could be used in future studies in order to provide consistent methodologies when investigating re‐treatment of MTA‐filled canals in extracted teeth.

There are several limitations to this study. First, due to the use of transparent acrylic blocks, the study does not replicate a true clinical scenario. However, this approach allows standardisation of samples, but it may also underestimate the difficulty in re‐treating root canals filled with MTA in clinical settings. The acrylic blocks with a uniform canal volume and anatomy do not fully replicate the complexity of natural root canal anatomy. Anatomical features such as isthmuses and accessory canals pose significant challenges during re‐treatment procedures as they can harbour residual filling materials and they can facilitate bacterial entrapment leading to treatment failure. Furthermore, MTA does not adhere to the walls of the plastic root canal model, which also does not replicate the clinical situation where MTA adheres to the dentine walls, so this potentially makes it more difficult to remove. Secondly, the effects of the ultrasonic instruments and rotary files used alone were not tested in this study. However, the aim was to assess the acids rather than the instruments. Hence, separate groups using these instruments alone, or in conjunction with a neutral solution such as water or saline, were not included. Third, there was no blinding of the operator and the manner in which the acrylic blocks were manipulated during this study may also have affected the results as the blocks were manually held and rotated to aid with the visualisation of MTA removal. Whilst this may have facilitated access to the root canal space and instrumentation, it raises concerns about the consistency and uniformity of the re‐treatment process across samples, and this would not be possible clinically. However, this limitation should be considered in light of the primary aim of this study, which was to assess the effectiveness of the acids on MTA removal in an attempt to determine which acid could then be investigated in a more clinically relevant scenario. Fourthly, some of the plastic may have been removed from the canal walls during the re‐treatment procedures, especially if a ledge was created. This may have affected the measurements of the weight of the samples after MTA removal. Fifth, the size of the re‐treatment files was 20/07, but the canals had been previously prepared to a size 30/07. Hence, some MTA may have been inadvertently left within the apical part of the canal, especially beyond the canal curvature. However, since the apical part of the canal could not be fully negotiated in the majority of samples, this is likely to have had minimal effect on the results. The final limitation is that the use of acrylic blocks does not allow assessment of the effects of the acids on dentine. Previous research has demonstrated that acidic solvents, at specific concentrations, can induce erosion of dentinal tubules, potentially compromising dentine integrity and strength [22]. Thus, it becomes imperative for future studies to explore the impact of acid concentrations on the dentine.

Whilst the findings of this study may have implications for other HCSCs due to their similar properties, further investigations are warranted to specifically validate these findings for different materials. Replicating this study with other HCSCs, particularly root canal filling cements that are gaining popularity in clinical practice, would provide valuable insights into their re‐treatment characteristics and acid interactions. Comparative studies across multiple HCSCs could elucidate potential differences in re‐treatment outcomes and inform clinical decision‐making regarding the selection of materials for root canal fillings.

## 5 Conclusion

Although none of the acids tested were able to completely remove the MTA root canal fillings, 10% citric acid was generally more effective, and 10% hydrochloric acid was the least effective. Challenges in removing MTA beyond root canal curvatures persist, suggesting a need for improved techniques and tailored acid to enhance MTA dissolution.

## Author Contributions

All authors acknowledge significant contributions and agree with the manuscript.

## Disclosure

The authors have nothing to report.

## Conflicts of Interest

The authors declare no conflicts of interest.

## Data Availability

Data are available upon request to the authors.
